# Molecular cloning and tissue expression of the fatty acid-binding protein (*Es-FABP*) gene in female Chinese mitten crab (*Eriocheir sinensis*)

**DOI:** 10.1186/1471-2199-11-71

**Published:** 2010-09-16

**Authors:** Ya-Nan Gong, Wei-Wei Li, Jiang-Ling Sun, Fei Ren, Lin He, Hui Jiang, Qun Wang

**Affiliations:** 1 School of Life Science, East China Normal University, Shanghai 200062, China

## Abstract

**Background:**

Fatty acid-binding proteins (FABPs), small cytosolic proteins that function in the uptake and utilization of fatty acids, have been extensively studied in higher vertebrates while invertebrates have received little attention despite similar nutritional requirements during periods of reproductive activity.

**Results:**

Therefore, a cDNA encoding *Eriocheir sinensis *FABP (Es-FABP) was cloned based upon EST analysis of a hepatopancreas cDNA library. The full length cDNA was 750 bp and encoded a 131 aa polypeptide that was highly homologous to related genes reported in shrimp. The 9108 bp *Es-FABP *gene contained four exons that were interrupted by three introns, a genomic organization common among FABP multigene family members in vertebrates. Gene expression analysis, as determined by RT-PCR, revealed the presence of *Es-FABP *transcripts in hepatopancreas, hemocytes, ovary, gills, muscle, thoracic ganglia, heart, and intestine, but not stomach or eyestalk. Real-time quantitative RT-PCR analysis revealed that *Es-FABP *expression in ovary, hemocytes, and hepatopancreas was dependent on the status of ovarian development, with peak expression observed in January.

**Conclusions:**

Evidence provided in the present report supports a role of Es-FABP in lipid transport during the period of rapid ovarian growth in *E. sinensis*, and indirectly confirms the participation of the hepatopancreas, ovary, and hemocytes in lipid nutrient absorption and utilization processes.

## Background

Fatty acid-binding proteins (FABPs) are small (14-15 k Da) cytosolic proteins that bind non-covalently to hydrophobic ligands, primarily fatty acids [1]. Physiological roles of FABP include, but are not limited to, the uptake and utilization of fatty acids, intracellular targeting of fatty acids to specific organelles and metabolic pathways, and the protection of cellular structures from the detergent effects of fatty acids [2-4]. Similarity among FABP deduced amino acid sequences in vertebrates and invertebrates are generally low despite the highly conserved gene structure of four exons and three introns of variable size [5,6], with the exception of desert locust FABP3 [7] and zebrafish FAPB1a [8]. Tertiary structure is common among all FABP family members, and consists of ten antiparallel β-sheet strands that surround the ligand binding domain [9].

To date, 12 FABP isoforms have been identified in vertebrates [10]. While genes were originally named according to the initial tissue from which they were first isolated, *e.g*. Liver-type FABP (L-FABP), Intestinal-type FABP (I-FABP), Heart-type FABP (H-FABP), Adipocyte-type FABP (A-FABP), Epidermal-type (E-FABP), isoform expression among multiple tissues and differences in tissue distribution among FABP orthologs have resulted in the implementation of numeric nomenclature, such that FABP1 corresponds to L-FABP, FABP2 to I-FABP, FABP3 to H-FABP, FABP4 to A-FABP, and FABP5 to E-FABP [9,11].

FABPs bind a single ligand molecule [12], with the exception of FABP1 and FABP10, both L-FABPs capable of simultaneously binding two ligand molecules [13]. FABP1 was the first FABP isolated from liver [14], and functions in fatty acid uptake [15] and metabolic pathway allocation in vertebrates [16], lipoprotein production [17], and nuclear delivery of peroxisome proliferator activated receptor (PPAR) ligands that results in the modulation of targeted gene expression [18]. FABP10 was first isolated from chicken liver [13], and is more similar in sequence to ileal FABPs than mammalian FABP1. To date, the FABP10 gene and protein have only been identified in nonmammalian vertebrates [8].

While FABPs among vertebrates have been studied in detail for more than three decades, with more than 400 identified, only ≈40 FABPs have yet been identified in invertebrates [1,9] and no information concerning FABP is available for *Brachyura*. The Chinese mitten crab (*Eriocheir sinensis*), a commercially important species in South-East Asia, is widely farmed in China and has quickly become an important aquaculture species [19] that has been cultured in ponds, reservoirs and lakes since the 1990's [20]. Hepatopancreas is generally regarded as a major lipid storage organ analogous to the fat body in insects and adipose tissue and liver in vertebrates [21]. The hepatopancreas in crab is a midgut diverticulum involved in the synthesis and secretion of digestive enzymes, final food digestion, nutrient absorption, and lipid and carbohydrate metabolism [22]; further, it is also a sensitive indicator of lipid metabolism and nutritional status [23]. Previous studies have shown that energy stored in crab hepatopancreas is in preparation for the significant expenditure required during the early stages of reproduction [24]. In order to elucidate potential functions of the hepatopancreas during reproduction in *E. sinensis*, a nonnormalized hepatopancreatic cDNA library was constructed [25]. EST analysis and subsequent cloning revealed an *Es-FABP *unigene, the first FABP gene identified in *Brachyura*. In the present study, we isolated FABP, for the first time from hepatopancreas of the Chinese mitten crab, investigated seasonal expression with respect to reproductive stage, and explored the relationship between FABP expression level and ovary development in the Chinese mitten crab in order to provide further insights into reproduction, nutrition and development of the Chinese mitten crab for farming industry.

## Methods

### Tissues preparation

Healthy adult female crabs (*E. sinensis*) were purchased from Tongchuan aquatic product market, Shanghai, China. Crabs were placed in an ice-bath for 1-2 min until lightly anesthetized. Eleven tissues were collected, including hepatopancreas, gills, stomach, intestine, cranial ganglia, thoracic ganglia, hemocyte, heart, muscle, and ovary, frozen immediately in liquid nitrogen, and stored at -80°C until nucleic acid extraction.

Based on the ovarian classification of Xue et al [26], ovarian Stage III-1 (40-50 mm diameter), III-2 (60-90 mm diameter), and IV (92-100 mm diameter) specimens were collected from August to September, September to November, and December to March, respectively, for ovarian *Es-FABP *expression analysis. For the present study, ovarian stages were further sub-divided based upon the month of dissection: Stage III-1 (Aug), Stage III-1 (Sep); Stage III-2 (Sep), Stage III-2 (Oct), Stage III-2 (Nov); Stage IV (Jan). Collection of hepatopancreas and hemocyte specimens occurred concomitantly with ovarian staged specimens. Tissues were immediately frozen in liquid nitrogen and stored at -80°C until RNA extraction.

### Nucleic acid extraction

RNA was extracted using a RNA Extraction Kit (Axygen, USA) according to the manufacturer's protocol. RNA concentration and quality were estimated by spectrophotometry at an absorbance of 260 nm, (Eppendorf Biophotometer plus, Germany) and agarose gel electrophoresis (Bio-Rad PowerPacBasic, USA), respectively. Total RNA (200 ng) was reverse transcribed using the PrimeScript™ RT-PCR Kit (TaKaRa, Japan) for semi-quantitative RT-PCR or the SYBR PrimeScript™ RT-PCR Kit (TaKaRa, Japan) for real-time quantitative RT-PCR (qRT-PCR).

Chinese mitten crab genomic DNA was extracted from hepatopancreas using the Axyprep™ Multisource Genomic DNA Miniprep Kit (Axygen, USA) according to manufacturer's protocol. DNA concentration and quality was estimated by spectrophotometry at an absorbance of 260 nm (Eppendorf Biophotometer plus, Germany) and agarose gel electrophoresis (Bio-Rad PowerPacBasic, USA), respectively.

### EST analysis and full-length cDNA cloning

A cDNA library was previously constructed from the hepatopancreas of Chinese mitten crab, and 3355 successful sequencing reactions were obtained using a T3 primer [25]. BLASTx analysis of EST sequences revealed 20 ESTs with high identity to *FcFABP *in *Fenneropenaeus chinensis *(ACU82845), which could be assembled into a single contig [a representative 527 bp EST is reported as GenBank: FG358115]. Sequence of the assembled *Es-FABP *contig was confirmed from the 3' direction using a T7 primer (Table 1).

**Table 1:** Oligonucleotide primers used to amplify the *E. sinensis FABP *gene

Primer name	Sequence (5'-3')	Application
T7	TAATACGACTCACTATAGG	cDNA cloning
SP6	ATTTAGGTGACACTATAGAA	cDNA cloning
5'SP1	CCCACAGCACTGAGCCCAATC	Genome walking
5'SP2	TATTTCCCGGTGATGGACATGA	Genome walking
5'SP3	GGTAGAAACAGCGAGGGCAACT	Genome walking
3'SP1	CACCTCCGCCAAGATTGTTATT	Genome walking
3'SP2	CGCACTTGAAAGAACCATAGCAG	Genome walking
3'SP3	GCTTTATGAGATTCGTTTGCGTGAT	Genome walking
F-R2	CAGAAGATGTTACAAGACTAAAG	RT-PCR
F-S2	GAGTTGCCCTCGCTGTTTGCTAT	RT-PCR
F-R3	CGTGGTCTTGATGACGATGT	RealTime-PCR
F-S3	TGGCTCAGTGCTGTGGGGT	RealTime-PCR
Actin-R	CTCCTGCTTGCTGATCCACATC	RT & RealTime-PCR
Actin-S	GCATCCACGAGACCACTTACA	RT & RealTime-PCR

To verify cDNA sequence, a gene-specific primer pair, F-S2 (sense) and F-R2 (anti-sense) (Table 1), was designed by Primer Premier 5.0 based on the sequence of the assembled contig mentioned above. The PCR reaction was performed in a ABI 2720 Thermal Cycler in a total volume of 25 μl and contained 2.5 μl of 10 × PCR buffer (Mg^2+ ^plus), 2.0 μl of dNTPs mix (2.5 mM each), 0.5 μl of each primer (20 μM), 18.375 μl of RNase-free water, 0.125 μl (5 U/μl) of Taq polymerase (TaKaRa, Japan), and 1 μl cDNA (500 ng/μl) as template. The PCR conditions were as follows: 94°C for 5 min; 30 cycles of 94°C for 30 s, 58°C for 30 s, 72°C for 30 s; and 72°C for 10 min. Appropriately sized PCR products were gel-purified and ligated into a pGEM-T easy vector (Promega, USA) with T4 DNA ligase. Positive clones containing inserts of predicted size were sequenced using T7 and SP6 primers (Table 1).

### Cloning of the *Es-FABP *gene and associated 5', 3'-flanking regions

The *Es-FABP *genomic DNA sequence was obtained with a gene-specific primer pair (F-S1, F-R1; Table 1), which corresponded to the cloned cDNA sequence. The final PCR reaction was performed in a total volume of 25 μl as described above. The 5'and 3'-flanking regions were cloned using a Genome Walking Kit (TaKaRa, Japan) according to the manufacturer's protocol with the following primers: 5'SP1, 5'SP2, 5'SP3, 3'SP1, 3'SP2, and 3'SP3. Specific products were purified and ligated into a pGEM-T easy vector (Promega, USA) with T4 DNA ligase. Positive clones were sequenced using SP6 primers (Table 1) and a 3730XL DNA analyzer (Applied Biosystems, Foster City, CA). The positions of exons and introns were determined by spidey. http://www.ncbi.nlm.nih.gov/IEB/Research/Ostell/Spidey/

### Phylogenetic analysis

Es-FABP nucleotide and deduced amino acid sequences were compared to those reported for other organisms using the BLAST algorithm at the National Center for Biotechnology Information http://www.ncbi.nlm.nih.gov/ to assess sequence identity. FABP amino acid sequences from *E. sinensis *and representative taxa were retrieved from NCBI GenBank and analyzed using the ClustalW Multiple Alignment program http://www.ebi.ac.uk/clustalw/. The open reading frame (ORF) of the cloned *Es-FABP *cDNA was determined by the ORF Finder http://www.ncbi.nlm.nih.gov/gorf/. A neighbor-joining (NJ) phylogenetic tree was constructed using the MEGA software version 4.0 package (http://www.megasoftware.net/). The confidence values for phylogeny analysis were determined by 1000 bootstrap replicates and expressed as a percentage. Three-dimensional domain structure of Es-FABP was predicted by Protein existence Server. http://www.uniprot.org/manual/protein_existence.

### Semi-quantitative RT-PCR analysis

Total RNA was extracted and reverse transcribed as described above. A predicted FABP PCR amplicon of 500 bp was generated using the gene-specific primer pair F-R2 and F-S2 (Table 1). The constitutively expressed beta-actin (Actin-R and Actin-S) produced a 276 bp product and served as an internal control. FABP and beta-actin genes were run in two single PCR reaction, with same PCR conditions and cycle number. The PCR reaction was performed in a final volume of 25 μl and contained 2.5 μl 10 × PCR buffer (Mg^2+ ^Plus), 2 μl 10 mM dNTP mixture, 0.25 μl 20 mM each primer, 18.875 μl PCR-Grade water, 0.125 μl 5 U Ex Taq™ Hot Start Version (TaKaRa, Japan), and 1 μl cDNA as template. PCR conditions were as follows: 94°C for 5 min; 30 cycles of 94 °C for 30 s, 55°C for 30 s, 72°C for 1 min; and 72°C for 5 min. RT-PCR products were separated on 1.5% agarose gel with ethidium bromide and detected under a Gel Doc 2000 ultraviolet light (Tannon, China).

### SYBR Green real-time qRT-PCR analysis

Real-time qRT-PCR was performed in a C1000™ Thermal Cycler (BioRad CFX 96™ Real-Time System) according to the manufacturer's instructions. PCR conditions were as follows: 95°C for 30 s; 40 cycles of 95°C for 5 s, 60°C for 30 s, with an 0.1°C/s incremental increase from 60°C to 95°C. The final volume of each qRT-PCR reaction was 25.0 μl, and contained 12.5 μl 2 × SYBR Premix Ex Taq (TaKaRa, Japan), 0.5 μl diluted cDNA template, 11.0 μl PCR-Grade water, and 0.5 μl of each 20 uM primer (F-R3, F-S3 Table 1). Beta-actin fragments were amplified using the primer pair Actin-R and Actin-S, and served as a positive control. Samples were run in triplicate, and *Es-FABP *expression levels were calculated by the 2^-ΔΔCt ^comparative CT method [27]. Data are represented as triplicate mean ± SD (standard deviation) and presented as the n-fold difference relative to beta-actin.

### Statistical analysis

Statistical significance was determined by a one-way ANOVA and a posthoc Tukey test using SPSS 11.5 software. Significance was set at *P *<*0.05*.

## Results

### Cloning and identification of the *Es-FABP *cDNA

**Figure 1:**
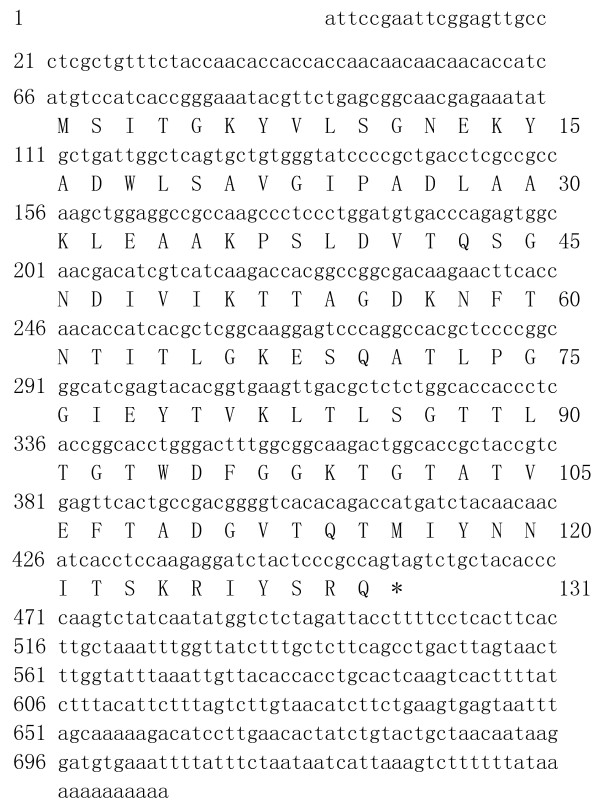
**Es-FABP nucleotide (above) and deduced amino acid (below) sequences**. Nucleotides numbering was initiated at the first base pair at the 5' end. Amino acid numbering began with the first in-frame methionine. The stop codon is marked by an asterisk. The polyadenylation signal (AATAA) is enclosed by a black ellipse. The *Es-FABP *sequence was submitted to GenBank [GenBank: GU568242].

A full length FABP cDNA (*Es-FABP*) was isolated from the hepatopancreas of a female Chinese mitten crab [GenBank: GU568242]. The full length cDNA (750 bp) contained a 393 bp open reading frame (ORF), which encoded a putative 131 amino acid FABP protein, a 65 bp 5'-untranslated region (UTR), and a 282 bp 3'-UTR (Fig. 1). A single polyadenylation signal (AATAA) was observed 719 bp upstream of the 12 bp poly (A) tail. Analysis of the *Es-FABP *cDNA sequence using BLASTX revealed significant sequence similarity to other FABPs sequences included in the National Center for Biotechnology Information database (NCBI, http://www.ncbi.nlm.nih.gov/BLAST/).

### Genomic sequence and structure of *Es-FABP*

**Figure 2:**
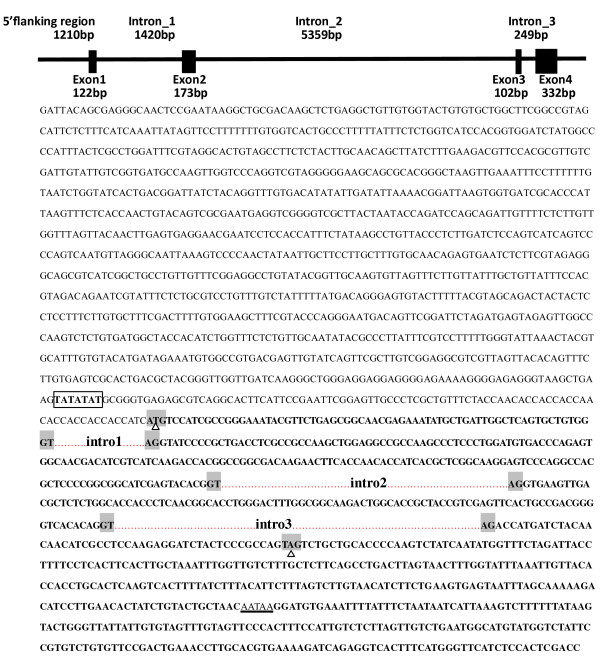
**Genomic organization and nucleotide sequence of *Es-FABP***. The coding sequence is in bold italics, and start and stop codons are shaded with "△" indicate their position. Intron sequences are represented by a dotted line. Exon/intron splice junctions (gt/ag) are shadowed. The 5'flanking region of the *Es-FABP *gene is shown in capital letters, and a putative TATA box enclosed by a black rectangle. The polyadenylation signal sequence AATAA is indicated by a black straight line.

**Figure 3:**
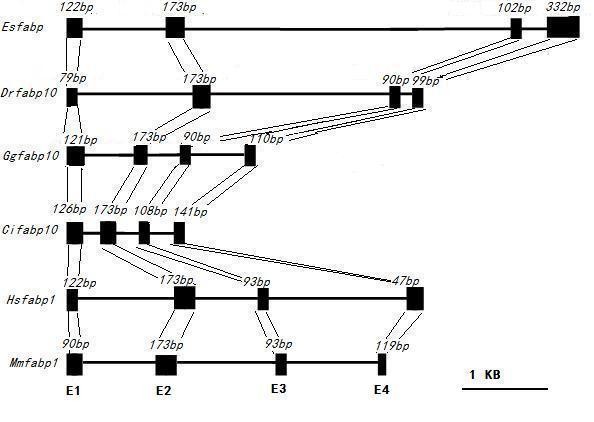
**The positions of exons and introns were determined by Spidey**. Alignment shows the following sequences: *Danio rerio *(*Drfabp10*) [GenBank accession no: NC_007127]; *Gallus gallus *(*Ggfabp10*) [NC_006110]; *Ciona intestinalis *(*Cifabp10*) [LOC100183372]; *Homo sapiens *(*Hsfabp1*) [NC_000002]; *Mus musculus *(*Mmfabp1*) [NC_000072]. Exons (E1, E2, E3, E4) are shown as blocks and introns as lines. The length of each exon is shown above the blocks. The size of each exon and intron is approximately represented by the length of blocks and lines. Intron scale bar:1000 basepairs (1 kb).

The *Es-FABP *gene was 9108 bp in length and was comprised of four exons interrupted by three introns (Fig. 2) [GenBank: GU568243]. Typical intron-exon junction structures with donor and acceptor (GT and AG) dinucleotide sequences were conserved in *Es-FABP*. *Es-FABP *genomic and cDNA sequences exhibited 99% similarity. The 1201 bp 5' anking upstream region contained a putative TATA box, but no binding motifs were identified. Comparison of the exon/intron organization of the *FABP *genes from *Danio rerio *(*fabp10*), *Gallus gallus *(*fabp10*), *Ciona intestinalis *(*fabp10*), *Homo sapiens *(*fabp1*), *Mus musculus *(*fabp1*) (Fig. 3). The positions of exons and introns were determined by an mRNA-to-genomic alignment using Spidey.

### Homology Analysis of Es-FABP

**Figure 4:**
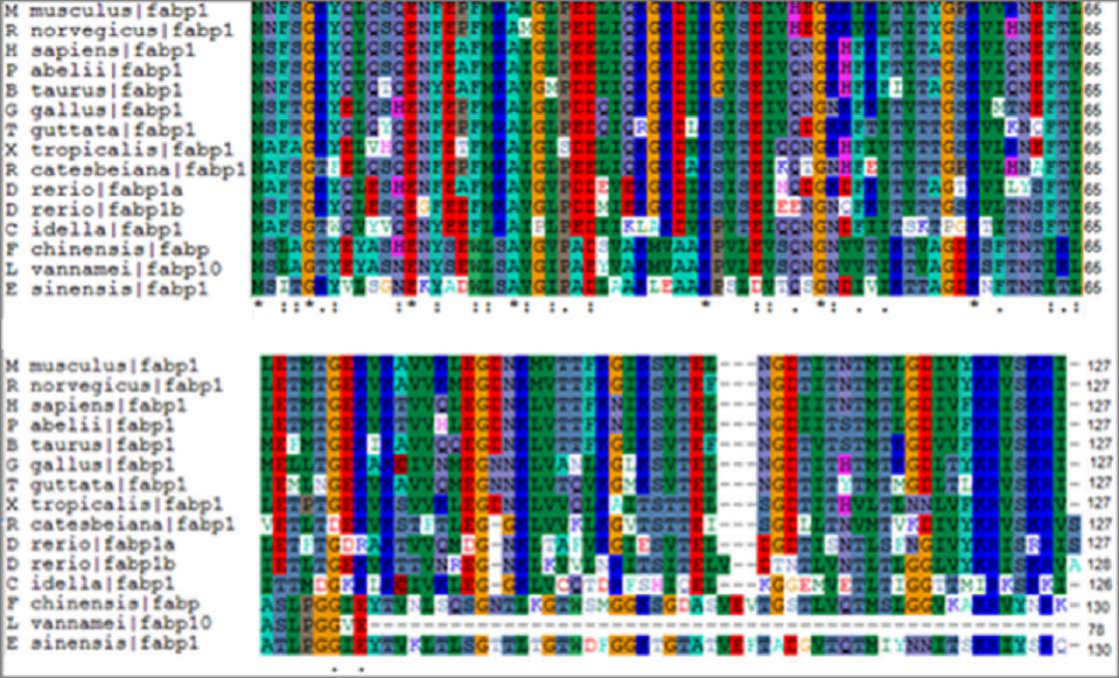
**ClustalX alignment of vertebrate and invertebrate FABP amino acid sequences**. Alignment shows the following sequences: *Mus musculus *(M_musculus, FABP1) [GenBank: NP_059095]; Rattus norvegicu*s *(R_norvegicus, FABP1) [NP_036688]; *Homo sapiens *(H_sapiens, FABP1) [NP_001434]; *Pongo abelii *(P_abelii, FABP1) [NP_001125017]; *Bos Taurus *(B_Taurus, FABP1) [NP_787011]; *Gallus gallus *(G_gallus, FABP1) [NP_989523]; *Taeniopygia guttata *(T_guttata, FABP1) [XP_002188068]; *Xenopus tropicalis *(X_tropicalis, FABP1) [NP_001116883]; *Rana catesbeiana *(R_catesbeiana, FABP1) [ACO51701]; *Danio rerio *(D_rerio, FABP1a) [NP_001038177]; *Danio rerio *(D_rerio, FABP1b) [NP_001019822]; *Ctenopharyngodon idella *(C_idella, FABP1) [ABW38784]; *Fenneropenaeus chinensis *(F_chinensis, FABP) [ACU82845]; *Litopenaeus vannamei *(L_vannamei, FABP10) [ABD65306]; *Eriocheir sinensis *(E_sinensis, FABP) [GU568242].

The identity of Es-FABP and other representative vertebrate and invertebrate deduced amino acid FABP sequences was explored via multiple sequence alignment using ClustalX (Fig. 4) [28]. Overall percent identify was high among Es-FABP and other reported invertebrate sequences, with 56% identity to *Fenneropenaeus chinensis *and 62% identity to *Litopenaeus vannamei*. Overall percent identity of Es-FABP and representative higher and lower vertebrates was substantially lower, with 21% identity to *Ctenopharyngodon idella*; 22% identity to *Sparus aurata*; 27% identity to *Xenopus tropicalis*; 28% identity to *Rattus norvegicus*; 29% identity to *Homo sapiens*, *Mus musculus*, *Danio rerio*, *Taeniopygia guttata, Rana catesbeiana, Pongo abelii*; 30% identity to *Bos Taurus*; 32% identity to *Gallus gallus*.

**Figure 5:**
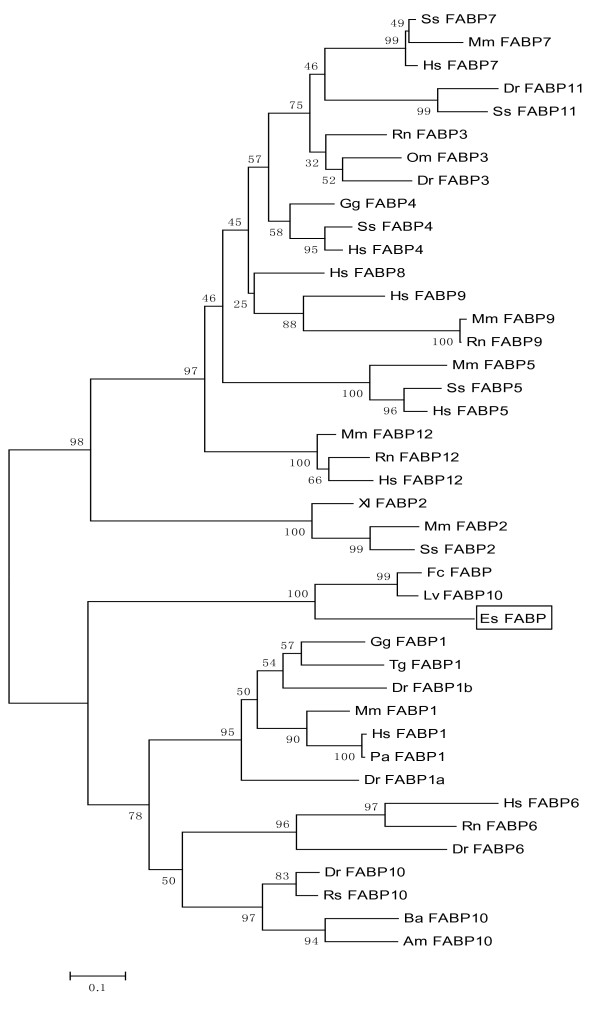
**Neighbor-Joining phylogenetic tree of representative vertebrate and invertebrate FABP amino acid sequences**. Bootstrap values supporting branch points are expressed as the percentage of 1000 replicates. The following organisms with FABP GenBank reported sequences were included in the analysis: *A. mexicanum *(Am-FABP10) [GenBank: P81400.2]; *B. arenarum *(Ba-FABO10) [P83409]; *D. rerio *(Dr-FABP1b) [AAZ08576], Dr-FABP1a [NP_001038177], Dr-FABP3 [NP_694493], Dr-FABP6 [ACD37360], Dr-FABP10 [AAI64928], Dr-FABP11 [NP_001004682]; *F. chinensis *(Fc-FABP) [ACU82845]; *G. gallus *(Gg-FABP1) [NP_989523], Gg-FABP4 [NP_989621]; *H. sapiens *(Hs-FABP1) [NP_001434], Hs-FABP4 [NM_001442], Hs-FABP5 [NP_001435], Hs-FABP6 [AAH22489], Hs-FABP7 [CAG33338], Hs-FABP8 [NP_002668], Hs-FABP9 [NP_001073995], Hs-FABP12 [NP_001098751]; *L. vannamei *(Lv-FABP10) [ABD65306]; *M. musculus *(Mm-FABP1) [NP_059095], Mm-FABP2 [AAS00550], Mm-FABP5 [NP_034764], Mm-FABP7 [CAJ18607], Mm-FABP9 [NP_035728], Mm-FABP2 [NP_083586]; *O. mykiss *(Om-FABP3) [NP_001118185]; *P. abelii *(Pa-FABP1) [NP_001125017]; *R. norvegicus *(Rn-FABP3) [NP_077076], Rn-FABP6 [NP_058794], Rn-FABP9 [NP_074045], Rn-FABP2 [NP_001128086]; *R. sapo *(Rs-FABP10) [P80856]; *S. scrofa *(Ss-FABP2) [NM_001031780], Ss-FABP4 [NM_001002817], Ss-FAPP5 [NP_001034835], Ss-FABP7 [AAY17257], Ss-FABP11 [CAM58515]; *T. guttata *(Tg-FABP1) [XP_002188068]; *X. laevis *(Xl-FABP2) [NM_001085877].

A NJ phylogenic tree was constructed based on reported FABPs amino acid sequences using MEGA4.0 software (Fig. 5). The reliability of the branching was tested using the bootstrap resampling (with 1000 pseudo replicates) technique. Two distinct sister groups were observed, with tree topology in agreement with traditional taxonomic relationships. The first group contained vertebrate FABP1, FABP6 and FABP10 sequences, as well as Es-FABP and shrimp FABPs. The second group contained vertebrate FABP2, FABP3, FABP4, FABP5, FABP7, FABP8, FABP9, FABP11, and FABP12 sequences.

A homology model of Es-FABP was predicted in SWISS-MODEL database http://swissmodel.expasy.org/ revealed conservation of tertiary structure, with the 10 anti-parallel β-strands forming a barrel and a clamshell-like structure.

### Tissue distribution and reproductive dependence of *Es-FABP *expression

**Figure 6:**
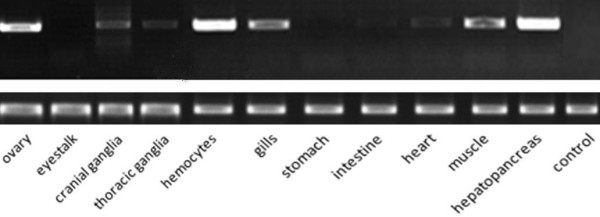
**Tissue distribution of *Es-FABP *expression as determined by semi-quantitative RT-PCR analysis**. *Es-FABP *RT-PCR and beta-actin results are displayed in the upper and lower panels, respectively. Appropriately sized amplicons were observed in RNA extracts from *E. sinensis *hepatopancreas, hemocytes, ovary, muscle, gills, cranial ganglia, thoracic ganglia, heart, and intestine, while expression was nondetectable in stomach, eyestalk, and the negative control (no template).

Semi-quantitative RT-PCR and real-time qRT-PCR were employed to investigate the distribution of *Es-FABP *mRNA in different tissues as well as to assess expression during the seasonal female reproductive cycle. As determined by RT-PCR, *Es-FABP *was widely distributed with high and detectable expression levels observed in hepatopancreas, ovary, hemocytes, and gills, muscle, cranial ganglia, thoracic ganglia, heart, intestine, respectively; while expression was nondetectable in stomach and eyestalk (Fig. 6).

**Figure 7:**
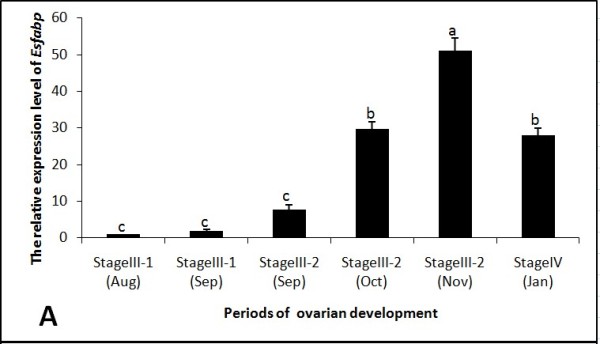
**Ovarian *Es-FABP *expression was influenced by the period of reproductive activity, as determined by real-time qRT-PCR**. *Es-FABP *expression, normalized to beta-actin, was quantified in mitten crab ovaries collected during the stages of rapid ovarian maturation: Stage III-1(Aug), Stage III-1(Sep), Stage III-2 (Sep), Stage III-2 (Oct), Stage III-2 (Nov), and Stage IV (Jan). Bars represent the triplicate mean ± S.E. from three individuals (n = 3). Bars with different letters differed significantly (P < 0.05).

**Figure 8:**
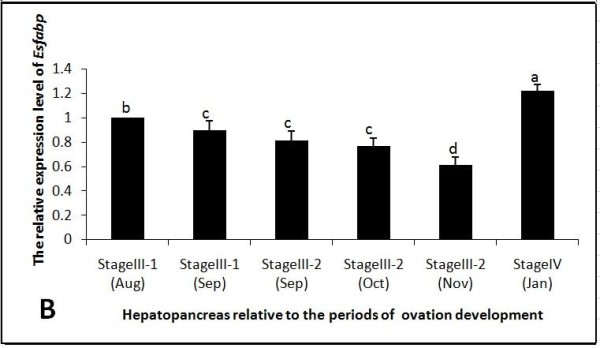
**Hepatopancreatic *Es-FABP *expression was influenced by the period of reproductive activity, as determined by real-time qRT-PCR**. *Es-FABP *expression, normalized to beta-actin, was quantified in mitten crab ovaries collected during the stages of rapid ovarian maturation: Stage III-1(Aug), Stage III-1(Sep), Stage III-2 (Sep), Stage III-2 (Oct), Stage III-2 (Nov), and Stage IV (Jan). Bars represent the triplicate mean ± S.E. from three individuals (n = 3). Bars with different letters differed significantly (P < 0.05).

**Figure 9:**
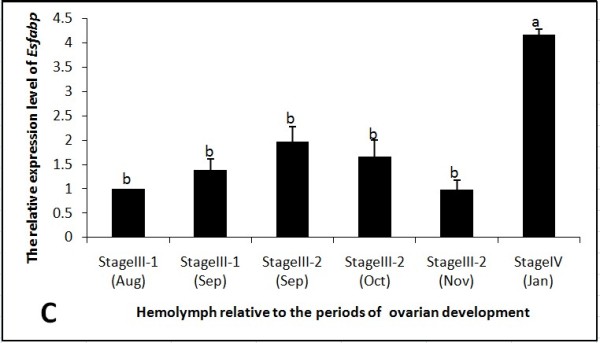
**Hemocytes *Es-FABP *expression was influenced by the period of reproductive activity, as determined by real-time qRT-PCR**. *Es-FABP *expression, normalized to beta-actin, was quantified in mitten crab ovaries collected during the stages of rapid ovarian maturation: Stage III-1(Aug), Stage III-1(Sep), Stage III-2 (Sep), Stage III-2 (Oct), Stage III-2 (Nov), and Stage IV (Jan). Bars represent the triplicate mean ± S.E. from three individuals (n = 3). Bars with different letters differed significantly (P < 0.05).

As determined by real-time qRT-PCR, beta-actin normalized *Es-FABP *expression in ovary increased rapidly and significantly from Stage III-1 (Aug-Sep) to reach peak expression during Stage III-2 (Nov) (Fig. 7). *Es-FABP *ovarian expression then decreased significantly through Stage IV (Jan). Hepatopancreatic expression decreased gradually from Stage III-1 (Aug) to Stage III-2 (Nov) and then increased significantly in Stage IV (Jan) (Fig. 8). *Es-FABP *expression in hemocytes did not differ significantly from Stage III-1 (Aug) through Stage III-2 (Nov), with a sharp peak observed during Stage IV (Jan) (Fig. 9).

## Discussion

FABP gene family members are evolutionarily conserved, with extensive and interdependent functions in the regulation of fatty acid oxidation/metabolism [9]. Although much research has focused on the classification, structure and function of vertebrate FABPs, these proteins remain unidentified in many invertebrate species. The current study reports the cloning and gene structure of a FABP family member in *E. sinensis*, *Es-FABP*, and presents evidence of functionality in fatty acid metabolism.

The full-length *Es-FABP *cDNA reported in the present paper encoded a putative FABP protein of 131 amino acids, which falls within the size range of other reported FABPs (127-136 amino acids) [29]. The ClustalX alignment of Es-FABP and fourteen other reported vertebrate and invertebrate FABP sequences revealed high identity among invertebrate sequences (56-62%) although percent identity remained lower when comparing within invertebrate and mammalian taxa (79-90%) [9]. Three-dimensional homology modeling revealed that several key tertiary structures of Es-FABP were similar to those of vertebrate FABP, such as the 10 anti-parallel β-strands, their resultant barrel with a clamshell-like structure, and the barrel cap comprised of a pair of α-helices, which enclose the FABP lipid-binding site cavity [30].

The cloned *Es-FABP *gene was 9108 bp in length, and was comprised of four exons interrupted by three introns, a conserved genomic organization among both species [31,32] and FABP superfamily members. Comparison of the exon/intron organization of the *FABP *genes revealed that, the genes are organized in four exons and three introns located in conserved positions, although varying markedly in size. And the second intron of *Es-FABP *was substantially longer than reported for other species. In general, while large introns are not unusual in organisms with very large genomes, they are less common among heavily transcribed genes, like the locust *FABP *[7]. Long introns might be favored because they enhance recombination, thus introducing mutations in adjacent exons [33] or intra- and inter- gene recombination [34], all of which would be subjected to selection pressures.

Phylogenetic analysis revealed two distinct sister groups within our constructed NJ tree as previously reported [8,35]: (1) group 1 contained FABP1, 6, 10, and (2) group 2 contained FABP2, 3, 4, 5, 7, 8, 9, 11, 12. Schaap *et. al*. estimated that FABP1, FABP6 and FABP10 diverged from a common ancestral gene ~679 million years ago (mya) [6]. FABP6 is an ileal-type FABP [36], while FABP1 and FABP10 are paralogs, and are thus evolutionarily related liver-type FABPs, which possibly arose by a gene duplication event [8]. Es-FABP segregated within group 1, clustering in close proximity to Fc-FABP10 and LvFABP10. ClustalX alignment results suggest that Es-FABP shares a high percent identity with FABP10. Based on collective phylogenetic evidence, we hypothesize that the Es-FABP we cloned from hepatopancreas is a FABP10.

We hypothesized that *Es-FABP *tissue expression profiles may provide useful cues when speculating gene function. In the present study, *Es-FABP *transcripts were primarily detected in hepatopancreas, hemocytes, and ovary. The hepatopancreas is generally regarded as a major storage organ of lipids, which are transported to reproductive organs and tissues during a period of reproductive activity [24]. Interestingly, the crustacean ovary contains a high lipid content compared to other organs [37,38], which is a key nutrient required for ovarian development and appropriate egg hatching rate and larval survival [38-41]. Further, the crustacean ovary is rich in long-chain poly unsaturated fatty acids (PUFA) [42]. As intracellular transporters of PUFA, FABP may serve as mediators of their physiological function, availability and access to intracellular targeted systems [10]. The high *Es-FABP *expression levels observed in these organs suggest that lipid nutrients, especially fatty acids, are transported from the hepatopancreas to the ovary in great supply via *Es-FABP *expressing hemocytes during the stage of rapid ovarian development. Es-FABP transported lipids are then stored in the ovaries in preparation for the significant and impendent energy expenditure required during reproduction [21].

Real-time qRT-PCR analysis revealed *Es-FABP *expression levels were dependent on the stage of ovarian development in all three organs. Fatty acids (FA) in general, including the ubiquitous palmitic and oleic acids, can regulate gene expression at the transcriptional level [43,44]. Furthermore, long-chain FA and long-chain dicarboxylic acids can act as inducers of *FABP1 *expression [45,46], as tissue FABP content is related to the rate of FA uptake and/or utilization [47-49]. Similarly, manipulations altering the rate of tissue FA metabolism appear to be associated with concomitant changes in FABP content [50]. Therefore, a direct or indirect feedback loop between FA and FABP may exist. In the present study, ovarian *Es-FABP *expression increased with the developmental progression of the ovary, mirroring lipid nutritional requirements. The ovary requires substantial accumulation of lipids, especially fatty acids, during developmental stages [51-53]. Interestingly, the rapid increase in ovarian lipid deposition was associated with a gradual decline in *Es-FABP *expression in hepatopancreas, suggesting hepatopancreas-mediated lipid transport may be occurring during ovarian development. Determining the existence or absence of a hepatopancreas-ovary feedback loop requires further study.

## Conclusions

In conclusion, evidence provided in the present report supports a role of Es-FABP in lipid transport during the period of rapid ovarian growth in *E. sinensis*, and indirectly confirms the participation of the hepatopancreas, ovary, and hemocytes in lipid nutrient absorption and utilization processes. However, further study concerning Es-FABP's specific mechanistic role in the process of lipid transport is both required and warranted.

## Authors' contributions

YNG carried out the molecular studies, participated in the gene cloning, sequence alignment and drafted the manuscript. WWL participated in the phylogenetic analysis, semi-quantitative RT-PCR and SYBR Green real-time qRT-PCR analysis. JLS participated in the tissue preparation. FR participated in the nucleic acid extraction. LH participated in the statistical analysis. HJ provided EST sequences. QW conceived of the study, and participated in its design and coordination. All authors read and approved the final manuscript.
